# Affinity-Bead-Mediated Enrichment of CD8+ Lymphocytes from Peripheral Blood Progenitor Cell Products Using Acoustophoresis

**DOI:** 10.3390/mi7060101

**Published:** 2016-06-09

**Authors:** Anke Urbansky, Andreas Lenshof, Josefina Dykes, Thomas Laurell, Stefan Scheding

**Affiliations:** 1 Department of Biomedical Engineering, Lund University, 221 00 Lund, Sweden; andreas.lenshof@bme.lth.se; 2 Department of Laboratory Medicine, Lund Stem Cell Center, Lund University, 221 00 Lund, Sweden; josefina.dykes@med.lu.se (J.D.); stefan.scheding@med.lu.se (S.S.); 3 Department of Clinical Immunology and Tranfusion medicine, University and Regional Laboratories, 221 85 Lund, Sweden; 4 Department of Biomedical Engineering, Dongguk University, 04620 Seoul, Korea; 5 Department of Hematology, University Hospital Skåne, 222 41 Lund, Sweden

**Keywords:** acoustophoresis, ultrasound, CD8 lymphocytes, magnetic-beads, cell sorting, PBPC, peripheral blood progenitor cells

## Abstract

Acoustophoresis is a technique that applies ultrasonic standing wave forces in a microchannel to sort cells depending on their physical properties in relation to the surrounding media. Cell handling and separation for research and clinical applications aims to efficiently separate specific cell populations. Here, we investigated the sorting of CD8 lymphocytes from peripheral blood progenitor cell (PBPC) products by affinity-bead-mediated acoustophoresis. PBPC samples were obtained from healthy donors (*n* = 4) and patients (*n* = 18). Mononuclear cells were labeled with anti-CD8-coated magnetic beads and sorted on an acoustophoretic microfluidic device and by standard magnetic cell sorting as a reference method. CD8 lymphocytes were acoustically sorted with a mean purity of 91% ± 8% and a median separation efficiency of 63% (range 15.1%–90.5%) as compared to magnetic sorting (purity 91% ± 14%, recovery 29% (range 5.1%–47.3%)). The viability as well as the proliferation capacity of sorted lymphocytes in the target fraction were unimpaired and, furthermore, hematopoietic progenitor cell assay revealed a preserved clonogenic capacity post-sorting. Bead-mediated acoustophoresis can, therefore, be utilized to efficiently sort less frequent CD8+ lymphocytes from PBPC products in a continuous flow mode while maintaining cell viability and functional capacity of both target and non-target fractions.

## 1. Introduction

Hematopoietic stem cell transplantation (HSCT) is an established therapy for hematological malignancies and other diseases. Potential stem cell sources are bone marrow, peripheral blood and umbilical cord blood. Over the last two decades peripheral blood progenitor cells (PBPC), which are collected after mobilization treatment, have replaced bone marrow as the main stem cell source for transplantations [1]. 

A standard transplant contains a variety of different cells including stem and progenitor cells as well as different lymphocyte populations [2,3,4], and it has been shown that an optimal graft composition of the transplant is crucial for the transplantation outcome [5]. For example, CD8 depletion in nonmyeloablative allogeneic stem cell transplantation decreased graft-*versus*-host disease (GVHD) while preserving engraftment and the graft-*versus*-leukemia (GVL) effect [6]. Other groups have shown that a higher CD8+ cell dose in myeloablative as well as nonmyeloablative allogeneic HSCT correlates with better T cell engraftment, improved freedom from disease progression and overall survival [7,8]. Therefore, graft processing methods have been developed aiming to provide an optimized transplant by enrichment or depletion of certain cell types. Following collection of chemotherapy- and/or hematopoietic growth factor–mobilized peripheral blood stem cells by large-volume leukapheresis [9], further processing of PBPC products is nowadays usually performed by large-scale magnetic cell sorting (MACS) [10,11]. Microfluidic approaches to separate lymphocyte subpopulations from clinical samples in continuous flow-based magnetic separations have not been reported. However, studies investigating the isolation of tumor cell lines spiked in blood have demonstrated high recoveries as well as reasonable purities [12]. Fluorescence-activated cell sorting (FACS) is also an option which, however, is limited by extensive processing times when sorting clinically relevant cell numbers and difficulties in complying with current good manufacturing practices (GMP) regulations [13,14]. 

As an alternative cell handling and sorting tool, acoustophoresis has gained increased attention for preclinical-scale cell sorting with a strong developmental potential towards later clinical applications [15,16]. Acoustophoresis utilizes an ultrasonic standing wave generated in a microchannel commonly of a width corresponding to half a wavelength [17]. The standing wave generates a pressure node in the center of the channel and a pressure anti-node at the sidewall, and consequently, the acoustic radiation forces will induce a movement of suspended particles either to the pressure node or the anti-node, depending on physical properties such as size, density and compressibility in relation to the suspending medium. Typically, denser particles such as cells or beads move to the pressure node in aqueous systems while less dense particles such as lipid particles are focused towards the pressure anti-node [18]. The magnitude of the radiation force increases with the cube of the particle radius and, therefore, larger particles move faster compared to smaller particles with the same acoustic properties [19]. Acoustophoresis, as a continuous laminar flow-based and easy-to-handle separation method, has been shown not to affect cell viability or functional capacity [20,21,22], and thus might offer an alternative for cell handling in clinical settings [15,23,24,25,26].

Recently, label-free separation of lymphocytes and granulocytes as well as platelets was demonstrated using microchip-based free-flow acoustophoresis [25,27]. Based on these results we developed a system to acoustically sort bead-labeled CD4 lymphocytes from peripheral stem cell products [28]. Bead-labeling was introduced to form bead-target cell complexes which have a higher net acoustic mobility than unbound cells and thus enable acoustophoretic discrimination of the target lymphocytes from the general lymphocyte population. Label-free acoustic separation of lymphocyte subpopulations is not possible due to the minute differences in acoustic properties, specifically in size.

In the current work, we investigated the performance of affinity-bead-mediated acoustophoresis to separate CD8+ cytotoxic T cells from PBPC products and optimized the sorting for this cell population, which is less frequent in the starting material compared to CD4+ cells. Following system optimization, we were able to increase the processing speed by a factor of two, as compared to our earlier work on acoustic separation of CD4+ cells [28], while still obtaining efficient separation with fully preserved functional capacity of sorted cells. Affinity-bead-mediated acoustophoresis can therefore be used to target and enrich specific cell populations in a continuously perfused microfluidic device. Furthermore, these data represent a step forward towards acoustic sorting of even rarer cell populations, such as peripheral blood stem cells and also open up the potential for simultaneous acoustic separation of multiple cell populations.

## 2. Materials and Methods

### 2.1. Ethical Statement

The use of patient and donor PBPC products in the current study was approved by the Regional Ethical Review Board at Lund University. Written informed consent was obtained from all participants involved in the study.

### 2.2. Sample Collection

Peripheral blood progenitor cell products were obtained by large volume leukapheresis performed with a Cobe Spectra (Terumo BCT, Lakewood, CO, USA), using the MNC program, version 7.0. Samples were collected after standard mobilization treatment of healthy donors (granulocyte colony-stimulating factor (G-CSF), Filgrastim, Sandoz, Novartis, Basel, Switzerland) and patients (protocol specific chemotherapy + G-CSF). On the day of leukapheresis, 1 mL of PBPC sample was removed from the collection bag and the mononuclear cell (MNC) fraction was isolated by Ficoll density gradient centrifugation for subsequent use in the experiments.

### 2.3. Labeling of CD8+ Cells with Affinity Beads

Superparamagnetic 4.5 µm polystyrene Dynabeads (Invitrogen Life Technologies, Thermo Fisher Scientific, Waltham, MA, USA) coated with primary monoclonal anti-CD8 antibody were used to label CD8+ cells in the MNC fraction. Magnetic beads used in this study were chosen for acoustic separation due to their commercial availability as well as to have a positive control of the separation procedure using a standard method, in this case magnetic sorting. In general, affinity-bead-mediated acoustophoresis can be performed with any kind of bead, as long as it has proper acoustic properties to distinguish bead-bound cells from unbound cells in the acoustic field.

In brief, 25 µL Dynabeads were added per 10^7^ cells/mL and incubated at 4 °C for 20 min under continuous agitation. The sample was split equally for tube-based magnetic separation on a DynaMag^TM^-15 magnet (Thermo Fisher Scientific) and acoustic separation on an acoustophoresis micro-chip in parallel (see below).

Following magnetic and acoustic separation, the isolated cells were incubated with DETACHaBEAD (10 µL per 10^7^ cells) at room temperature for 45 min under continuous agitation to release bound beads. The cell-bead mixture was exposed to a DynaMag^TM^-15 magnet and the supernatant containing released cells was transferred to a fresh tube. The beads were washed twice in 1 mL PBS + 2% fetal bovine serum (FBS) (Gibco Life Technologies, Thermo Fisher Scientific) and 0.6% acid-citrate-dextrose (ACDA) (Terumo BCT) to recover residual cells. Collected cells were washed by centrifugation at 400× *g* for 5 min, stained with Trypan blue (Gibco Life Technologies) for dead cell exclusion and counted using a Neubauer chamber.

### 2.4. Magnetic Cell Separation

Magnetic separation was performed according to manufacturer’s instructions (Dynabeads CD8 Positive Isolation Kit, Invitrogen Life Technologies). Bead-labeled cells were isolated using a DynaMag^TM^-15 magnet while non-labeled cells were removed by washing three times with 1 mL wash buffer (PBS with 2% FBS and 0.6% ACDA). Isolated cells were released from the magnet and re-suspended in wash buffer (100 µL/10^7^ cells).

### 2.5. Acoustophoresis Chip

**Figure 1:**
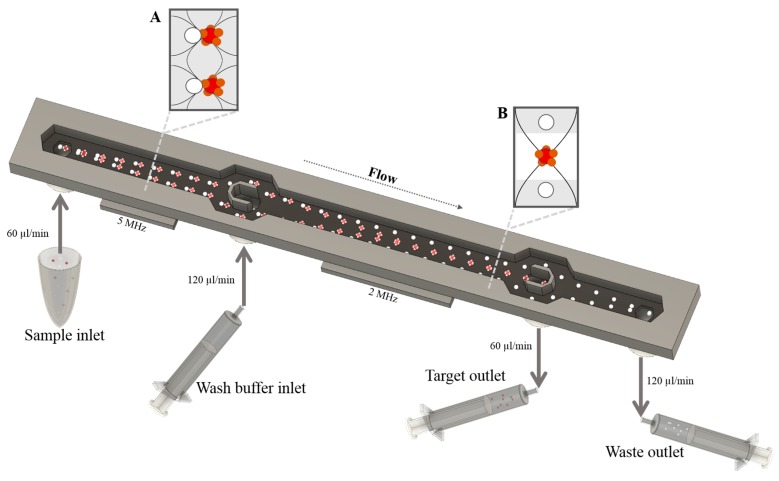
Schematic picture of the acoustophoretic chip design. The sample consisting of a mixture of bead-bound cells (red) and unbound cells (white) is injected into the pre-focusing channel through the sample inlet. In a first step particles are pre-focused into two parallel bands (**A**) using a 5 MHz piezoceramic transducer that drives a full wavelength resonance across the channel width, with two pressure nodes that superimpose with a half-wavelength resonance in the vertical direction. Following the flow direction, the particles are then bifurcated to each side of the wash buffer inlet where Ficoll is infused. Due to the pre-focusing step, cells enter the separation channel close to the channel walls and are prealigned in the width and height dimension of the channel, ensuring an identical starting position for the separation procedure and thereby maximizing the resolution of the separation. In the main separation channel, acoustic forces in an ultrasonic standing wave field with a pressure node in the center of the channel induce movement of cells and particles depending on their acoustophysical properties. Bead-bound cells are forced into the Ficoll buffer (**B**) and can be collected through the target outlet while unbound cells stay close to the channel wall and can be collected through the waste outlet.

A detailed description of the acoustophoresis chip design and fabrication process can be found in Augustsson *et al*. [26]. In brief, the structure of the microchannel was made by anisotropic wet etching in a silicon wafer using standard photolithography and anisotropic KOH etching, and sealed with a glass lid using anodic bonding. The chip consists of a sample inlet, leading to a pre-focusing channel (10 mm × 300 μm × 150 μm), a flow splitter that directs the flow to each side of a central fluid inlet, and a main separation channel (20 mm × 375 μm × 150 μm) ending in a trifurcation with one central outlet and a common outlet for the two side branches (Figure 1). Docking ports for fluidic tubing were glued to the inlets and outlets in form of silicon tubing with an inner diameter of one-sixteenth of an inch.

A piezoceramic transducer resonant at 5 MHz was glued underneath the pre-focusing channel, while a second piezoceramic transducer resonant at 2 MHz was attached underneath the main separation channel. Both transducers were driven by a dual channel function generator (AFG3022B, Tektronix, Beaverton, OR, USA), equipped with signal amplifiers (in-house build), and the voltage over each transducer was measured via a two-channel digital oscilloscope (TDS 1002, Tektronix). For visualization of the separation procedure a Nikon SMZ800 microscope (Nikon, Tokyo, Japan) was used.

### 2.6. Fluidic Setup and Sample Procedure

The flow through the chip was controlled by three syringe pumps (neMESYS, Cetoni GmbH, Korbußen, Germany). Two of the pumps were coupled to the chip outlets in withdrawal mode and connected to 1 mL plastic syringes (BD Plastipak, Becton Dickinson, Franklin Lakes, NJ, USA). The third pump was used to infuse Ficoll wash buffer (Histopaque-1077, Sigma-Aldrich, St. Louis, MI, USA) via a 5 mL glass syringe (Hamilton Bonaduz, Bonaduz, Switzerland) to the chip central inlet. 

Before sample processing, the system was flushed with PBS to evacuate air. The flow rate on each of the three exit branches was set to 60 μL/min and the wash buffer was infused at a flow rate of 120 μL/min. The bead-labeled PBPC sample (1 × 10^7^ MNC in 1 mL) was connected to the chip side inlet and entered into the pre-focusing channel at a net flow rate of 60 μL/min. Due to the pre-focusing step, cells are prealigned in the width and height dimension and enter the separation channel laminated towards the channel walls ensuring an identical starting position for the separation procedure and thereby enhancing the resolution of the separation [26]. The operational parameters such as frequency and voltage were set by visual inspection of the outlet trifurcation, showing an optimal focus of the bead-labeled cells into the central outlet while non-labeled cells were distributed to the side branches. Labeled cells in the center outlet (target fraction) and non-labeled cells in the side outlet (non-target fraction) were collected directly in the syringes. 

### 2.7. Flow Cytometric Analysis

A four-color flow cytometer (FACSCalibur, BD Biosciences, Becton Dickinson) was used to analyze the PBPC samples before and after magnetic and acoustic processing. Cells were spun down at 400× *g*, 4 °C for 5 min, re-suspended in PBS + 2% Gammanorm (Octapharma AG, Lachen, Switzerland) + 1% FBS and incubated with monoclonal antibodies for 35 min at 4 °C in the dark. Directly conjugated monoclonal antibodies used in different combinations in this study were: anti-CD3 fluorescein isothiocyanate (FITC) (clone SK7), anti-CD4 allophycocyanin (APC) (clone SK3), anti-CD8 phycoerythrin (PE) (clone SK1), anti-CD19 APC (clone HIB19), anti-CD34 PE (clone 581), anti-CD45 FITC (clone 2D1), anti-CD45 peridinin-chlorophyll protein (PerCP) (clone 2D1), and anti-CD56 PE (clone MY31), as well as corresponding isotype controls (all from BD Bioscience). Cells were washed by adding 1 mL FACS buffer (PBS, 1% BSA, 0.1% sodium azide (Sigma-Aldrich)), spun down for 5 min at 3000 rpm and re-suspended in FACS buffer. For dead cell exclusion 7-amino-actinomycin D (7-AAD, 200 µg/mL, Sigma-Aldrich) or propidium iodide (PI, 1 µg/mL, Sigma-Aldrich) was added to the cells. Data acquisition on the FACSCalibur was performed using the CellQuest software (BD Biosciences), recording 10,000 events and analyzing the data with FlowJo software (Tree Star Inc., Ashland, OR, USA).

### 2.8. In Vitro Cell Proliferation Assay

The proliferation capacity of isolated CD8+ cells in response to anti-CD3/anti-CD28 stimulation was evaluated by flow cytometry utilizing carboxyfluorescein diacetate succinimidyl ester (CFSE) staining. In brief, 1 × 10^6^ target cells were suspended in 1 mL pre-warmed PBS/0.1% BSA) and CellTrace CFSE-solution (Invitrogen Life Technologies) at a final concentration of 0.5 µM. Following 10 min of incubation at 37 °C, the staining was quenched by addition of five volumes of ice-cold culture medium (RPMI-1640 (Gibco Life Technologies), 10% human AB serum (Thermo Fisher Scientific)). Following 5 min incubation on ice, cells were washed three times by centrifugation at 400× *g* for 5 min. CFSE labeled CD8+ cells were cultured in duplicates at 15,000 cells per well in a 96-well flat bottom plate (TPP Techno Plastic Products) in a final volume of 200 µL culture medium. Cells were stimulated with anti-CD3 (5 µg/mL) and anti-CD28 (2 µg/mL) (eBioscience) in presence of 50 ng/mL IL-2 (Miltenyi Biotech) and incubated for up to four days (Thermo Forma Steri incubator, 37 °C, 5% CO_2_). At indicated time points CFSE fluorescence intensity distributions were measured by flow cytometry (FACSCalibur, CellQuest and FlowJo software) to analyze cell proliferation.

### 2.9. Hematopoietic Progenitor Cell Assay

Standard colony-forming cell assay using methylcellulose culture (MethoCult H4435 Enriched, Stemcell Technologies Inc., Vancouver, BC, Canada) was used to evaluate the hematopoietic progenitor cell content in PBPC samples and acoustic non-target fractions. Cells were plated at a concentration of 5000 cells/mL and incubated for 14 days at 37 °C and 5% CO_2_. Colony-forming units (CFU) were examined using a CK2 inverted microscope (Olympus, Tokyo, Japan) and counted based on standard criteria.

### 2.10. Statistical Analysis

Statistical tests were performed using GraphPad Prism 5.0 (GraphPad Software, San Diego, CA, USA). Using the paired or unpaired *t*-test, statistical significance was determined for *p* values ≤0.05.

## 3. Results

### 3.1. Enrichment of CD8+ Lymphocytes Using Affinity Bead Acoustophoresis

**Figure 2:**
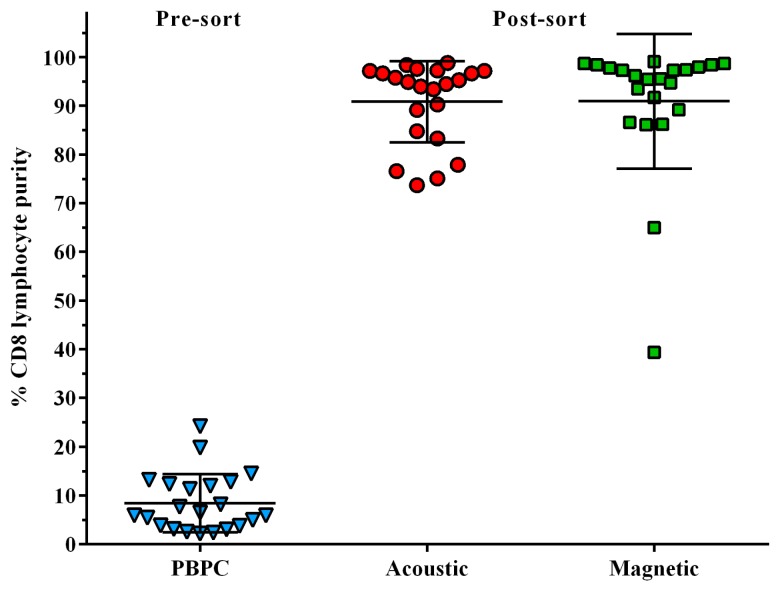
Frequency of CD8+ cytotoxic T cells in pre-sorted peripheral blood progenitor cell (PBPC) products and CD8+ purities following acoustic and magnetic separation post-sorted samples are shown. Both, acoustic and magnetic separation allowed effective enrichment of CD8+ cells. Data are presented as individual data points (triangles, circles, and quadrants) and corresponding means ± SD, *n* = 22.

The performance of affinity-bead-mediated enrichment of CD8+ lymphocytes from PBPC products using acoustophoresis was evaluated in comparison to standard magnetic cell sorting (Figure 2). Results from 22 samples (healthy donor *n* = 4, lymphoma *n* = 7, myeloma *n* = 8, multiple sclerosis *n* = 3) showed an efficient separation of targeted cells with a mean purity (±SD) of 90.9% ± 8.3% for acoustic sorting as compared to 90.9% ± 13.8% for magnetic sorting. In the magnetic separation, two samples had a purity of less than 65%, whereas for the corresponding acoustically-sorted samples purities of 94.5% and 97.2%, respectively, were reached. 

The median separation efficiency for acoustically sorted samples, as calculated by the ratio of CD8 cells in the target and non-target fraction, was 63.2% (15.1%–90.5%) in comparison to a median recovery of 28.6% (5.1%–47.3%) for standard magnetic separation as defined by the ratio of post-sorted and pre-sorted CD8 cells. Furthermore, the viability of sorted cells, as obtained with 7-AAD staining, was 97.6% ± 1.8% in acoustically sorted samples as compared to 98.3% ± 1.4% for magnetic sorting. 

### 3.2. Distribution of Leukocyte Subpopulations

**Figure 3:**
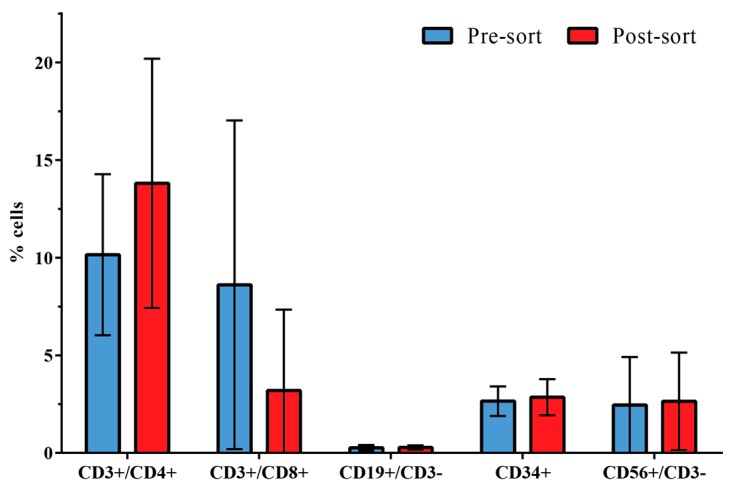
Flow cytometry analysis of the distribution of leukocyte subpopulations in the non-target fraction (side outlet) of acoustically sorted samples. Comparison of the mean (±SD) percentages of CD3+/CD4+, CD3+/CD8+, CD19+/CD3−, CD34+ and CD56+/CD3− cells in pre-sorted PBPC and post-sorted non-target samples (*n* = 3). Due to the removal of CD8+ cells from the sample and collection in the target fraction, a relative decrease of CD3+/CD8+ cells is observed in the non-target fraction compared to the input PBPC sample.

Flow cytometry analysis was chosen to reveal changes in the distribution of leukocyte subpopulations (*n* = 3) in pre-sorted PBPC samples compared to the non-target fraction after acoustic sorting (post-sort). As expected, the selective removal of CD8+ cells into the target fractions led to a relative increase of non-CD8+ cells in the non-target fraction as compared with the pre-sorted samples (Figure 3). 

Comparison of the mean (±SD) relative distribution between leukocyte subsets in the pre-sort *versus* post-sort sample showed the following changes: CD3+/CD4+ T helper cells, 10.15% ± 4.13% *versus* 13.81% ± 6.38%; CD3+/CD8+ cytotoxic T cells, 8.61% ± 8.42% *versus* 3.20% ± 4.14%; CD19+/CD3− B cells, 0.26% ± 0.15% *versus* 0.28% ± 0.11%; CD34+ hematopoietic stem and progenitor cells, 2.65% ± 0.76% *versus* 2.85% ± 0.92%; and CD56+/CD3− natural killer cells, 2.45% ± 2.46% *versus* 2.64% ± 2.50%. 

The low CD19+/CD3− B cell count is due to the fact that all three samples used for the leukocyte subset analysis were from patients with multiple myeloma, for whom a statistically significant decrease of the percentage of total CD19+ cells is observed compared with healthy controls [29].

### 3.3. CD3/CD28-Mediated T Cell Proliferation Capacity of Acoustically Sorted Cells Is Unimpaired

**Figure 4:**
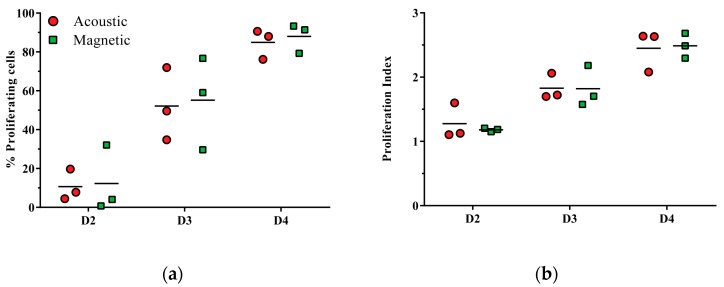
CD3/CD28-mediated T cell proliferation of acoustically and magnetically sorted CD8+ cytotoxic T cells. Cells were stimulated in the presence of anti-CD3/CD28 and proliferation was measured on days 2, 3 and 4 of culture using CFSE staining (*n* = 3). For each day the relative number of proliferating cells (**a**) as well as the proliferation index, *i.e.*, the average number of cell divisions all responding cells have undergone, are presented (**b**).

The proliferative response of acoustically and magnetically sorted CD8+ cytotoxic T cells stimulated with anti CD3/CD28 was evaluated after two, three and four days of culture (*n* = 3). The results show similar proliferation capacities of acoustically sorted cells compared to the magnetically sorted cells (Figure 4). The mean (±SD) relative number of proliferating cells for acoustically sorted cells *versus* magnetically sorted cells was: 10.7% ± 8% *versus* 12.3% ± 17.2% on day 2, 52.1% ± 18.7% *versus* 55.2% ± 23.8% on day 3, and 84.9% ± 7.7% *versus* 88.0% ± 7.6% on day 4.

In addition to the relative number of proliferating cells, the proliferation index, *i.e.*, the average number of cell divisions all responding cells have undergone, indicated no significant differences between acoustically and magnetically sorted cells. The proliferation index for acoustically *versus* magnetically sorted cells was 1.28% ± 0.28% *versus* 1.18% ± 0.03% on day 2, 1.83% ± 0.20% *versus* 1.82% ± 0.32% on day 3, and 2.45% ± 0.32% *versus* 2.49% ± 0.19% on day 4. 

### 3.4. Hematopoietic Progenitor Cell Colony-Forming Ability Is Unaffected by Acoustophoresis

**Figure 5:**
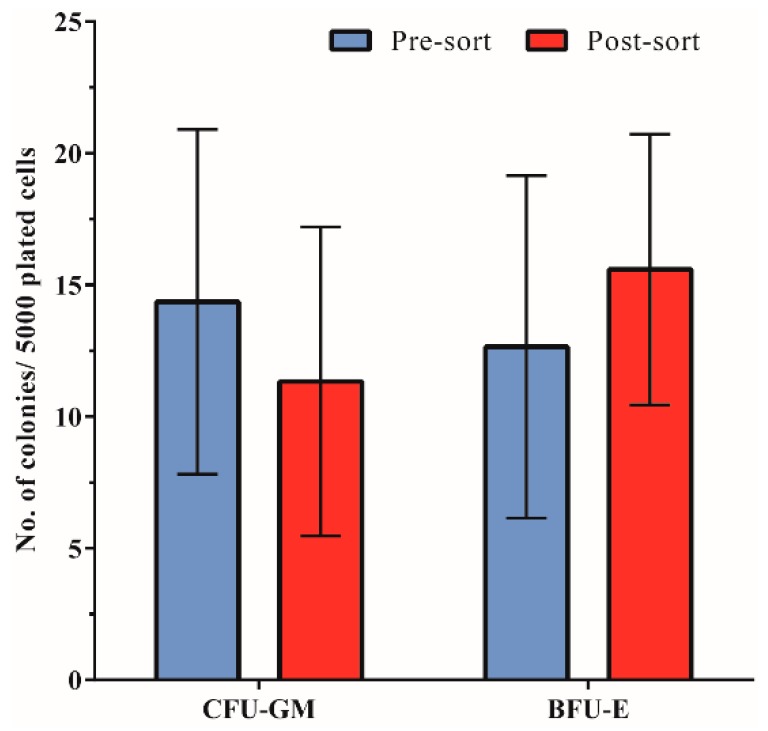
Hematopoietic progenitor cell colony-forming ability. The mean (±SD) number of granulocyte macrophage colony-forming units (CFU-GM) as well as erythroid burst-forming units (BFU-E) is shown for cells from pre-sorted (PBPC) and acoustically post-sorted (non-target) fractions (*n* = 4).

Colony-forming ability of hematopoietic progenitor cells as evaluated by standard methylcellulose assay revealed a preserved clonogenic capacity post-sorting (Figure 5). The mean (±SD) number of granulocyte macrophage colony-forming units (CFU-GM)/5000 cells was 14.6 ± 6.5 for PBPC samples (pre-sort) as compared to 11.3 ± 5.9 in the corresponding acoustic non-target fraction (post-sort). Correspondingly, there was no significant difference between the mean (±SD) number of erythroid burst-forming units (BFU-E)/5000 cells in the pre-sort (12.6 ± 6.5) *versus* post-sort samples (15.6 ± 5.1).

## 4. Discussion

Acoustophoresis, as a gentle microfluidic separation method, is based on acoustophysical properties such as size, density and compressibility of the particles in relation to the suspending medium. Previously, acoustophoretic label-free cell separations based on significant size differences have been shown for tumor cell enrichment from white blood cell samples [26], for blood lymphocyte and granulocyte separation [27], as well as for the removal of platelets from PBPC products [25].

In the current study, separation of lymphocyte populations from PBPC products was investigated. Similar acoustic properties of the different lymphocyte subpopulations make it impossible to realize a label-free sorting strategy using acoustic forces. Therefore, we used 4.5 µm affinity beads to label the cells of interest, thereby increasing the size as well as changing the acoustic contrast factor such that the bead-cell complex obtains an increased acoustic mobility as compared to non-labeled cells. However, due to the large variety in the cell diameter of lymphocytes (6–20 µm) [30,31,32] and the variable number of beads that can bind to a target cell, is it likely that the size of the bead-cell complexes partly overlaps with the non-labeled lymphocytes. We therefore also modified the medium properties of the central buffer to adjust the acoustic forces on the different particles to improve the discrimination of bead-bound cells *versus* non-bead-bound cells. This was performed by adjusting the density of the medium in the central inlet of the acoustophoresis chip, utilizing the known fact that the acoustic contrast factor and hence the resulting acoustic radiation force on a cell is dependent on the density difference *versus* the surrounding medium. The option to tune the net acoustic force on different cells in a sample was previously demonstrated by Peterson *et al*. by adjusting the density of the cell suspension buffer with cesium chloride [19].

**Figure 6:**
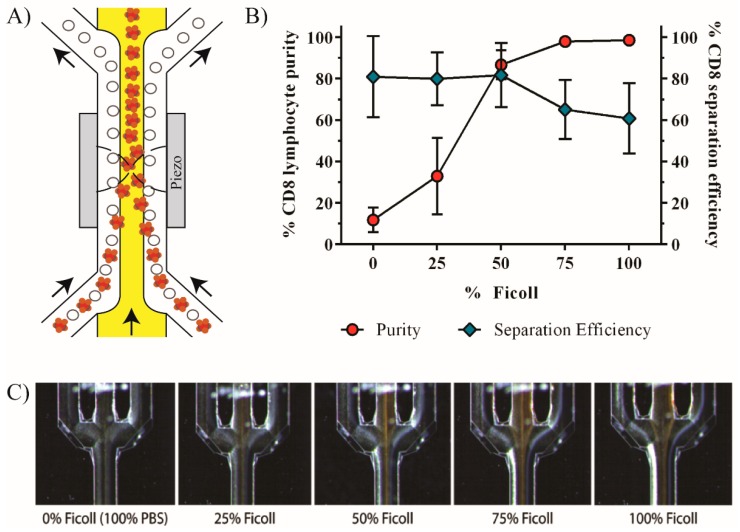
Purity and separation efficiency of acoustically sorted CD8+ cells in relation to the central buffer density. (**A**) Schematic drawing of the microfluidic device showing the effect of the high density central buffer. Bead-labeled cells (orange) and unlabeled cells (white) enter into the main separation channel. High density buffer infused in the central inlet (yellow) creates a barrier across which bead-labeled cells can be acoustically moved into the center stream and collected in the central outlet while unlabeled cells are not able to move into the high density buffer and thus exit the separation channel through the side outlet. Arrows indicate the flow direction; (**B**) Ficoll was diluted in PBS in different concentrations and used as central buffer. The purity of the acoustic separation as well as the separation efficiency was determined (*n* = 3); (**C**) The image sequence of the central outlet illustrates the effect on the acoustic separation at increasing levels of Ficoll in the central buffer. The increase of the central buffer density creates a barrier through which bead-labeled cells (orange) are able to move whereas unlabeled cells (white) are not and remain at the sidewall to be collected through the side outlet.

The beads used in our experiments have a density of 1.6 g/mL, whereas unbound cells have a lower density of 1.055–1.085 g/mL [33]. Using Ficoll with a density of 1.078 g/mL as a central buffer, an acoustophoretic barrier is created across which the bead-cell complex is able to migrate. Unbound cells, having a mean lower density than the central buffer, either cannot overcome this barrier or migrate very slowly in the higher density buffer. They can thus be isolated from the bead-cell population and collected at the side outlet of the acoustic chip (Figure 6). By optimizing the central buffer medium properties it became possible to sort CD8+ lymphocytes from mononuclear cells with high purity (90.9%) and efficiency (63.2%) into the center outlet. Analysis of lymphocyte subpopulations in the non-target fraction of the acoustically sorted sample showed a slight relative increase of CD4, CD19, CD34 as well as CD56 cells, which was due to removal of CD8 cells into the target fraction (Figure 3). The distribution of the populations is thus in the expected range. The viability and proliferation capacity of sorted cells were unimpaired, as was the colony-forming ability of hematopoietic stem and progenitor cells in the non-target fraction. These results are in good agreement with previously published data [15,22] and show that acoustophoresis is a promising sorting technology also for possible clinical applications where optimizing the cell composition is of importance.

The use of affinity microbeads in acoustophoretic applications has previously been shown to allow the capture of phage viral particles [34] and also to sort CD4 lymphocytes from PBPC products [28]. In this paper, we show the ability to sort a lymphocyte population which is considerably less abundant in the starting material as compared to CD4 cells, and our data clearly show that optimized acoustic sorting as described herein can be successfully applied also to the sorting of less frequent cell populations. Also, we were able to increase the throughput of the sample by a factor of two (600,000 cells/min, 60 µL/min sample flow), as compared to acoustic sorting of CD4 cells (300,000 cells/min, 30 µL/min sample flow), while maintaining high purity of the sorted sample and preserving functional capacity of both the target and non-target cells. Affinity-bead-mediated acoustophoresis can therefore be used to target and enrich specific cell populations in a continuously perfused microfluidic device. Furthermore, this is a promising step towards the sorting of rare CD34+ hematopoietic stem and progenitor cells, which make up around only 1% to 2% of the MNC collected from peripheral blood after mobilization treatment [35], as compared to 8.4% ± 5.8% CD8+ cells (own data).

Processing of PBPC products for clinical use is nowadays usually performed by large-scale magnetic cell sorting using the Miltenyi CliniMACS system (Miltenyi Biotec, Bergisch Gladbach, Germany). Here, the cells of interest are labeled with 50-nm-sized magnetic particles and are either depleted or positively selected using a magnetic field in an automated device. Magnetic sorting with the CliniMACS can be performed with high purities and recoveries of usually 95%–99% and 60%–70%, respectively [36,37,38], as well as high throughput of sample. The CliniMACS sorting process is a batch process and the separation is limited by the loading capacity of the magnetic column [39], whereas acoustophoresis offers a continuous flow-based separation procedure without limitation of the processed sample volume. However, processing of clinical-scale samples by acoustophoresis would require a substantial upscale of the throughput. When compared to the CliniMACS system, which is able to process samples at a speed of 1 mL/min [40], the throughput of the acoustophoresis microchip is much slower and would have to be increased by a factor of 10 to process PBPC at a clinical scale. Likely, this can be achieved by increasing the length of the separation channel and/or by using several microchannels in parallel [23]. However, the scope of the current work has been to show a proof of concept that matches the performance of clinical practice and an up-scaled system having clinical capacity is a next step in the technical development.

Our data show that the performance of acoustic separation is in the range of what is reported for other systems and was even superior compared to the control sorting experiments performed in parallel with the Dynabead system. Nevertheless, further improvements of the acoustic cell sorting system are of course desirable, and could be realized by optimization of the experimental setup, for example enhancing the separation stability of the acoustic setup, such as wash buffer modification, maximizing flow rates and acoustic energy in the channel, temperature and frequency control and implementation of a more stable pressure-driven flow control system as compared to syringe pumps. Augustsson *et al*. [41] reported a temperature dependence of the resonance frequency and acoustic energy in the microchannel and later implemented a thermostabilized system to enable long-term stable operation at a fixed resonance frequency [26]. Moreover, the beads used in the experiments were not optimized for acoustophoresis but rather chosen because they allowed a direct comparison with magnetic sorting. Different bead materials and sizes as well as optimization of the binding capacity therefore represent additional ways to potentially increase performance.

Acoustophoresis is an easy-to-handle separation technology and it provides an interesting alternative in the handling of PBPC products for research purposes, pre-clinical as well as clinical applications. The microfluidic device can easily be combined in line with additional up- and down-stream applications, for example combining the separation of target cells with direct preparation and analysis of the separated cells [24]. Also, the separation of multiple target fractions using different-sized beads in a suitable microfluidic chip design [19] is possible and offers the opportunity for simultaneous sorting of two or more cell types, a feature which cannot be realized with current magnetic sorting devices.

## 5. Conclusions

In this study we show that acoustophoresis of bead-labeled cells can be utilized to efficiently sort the CD8+ lymphocyte population from peripheral blood progenitor cell products. Optimization of the system substantially increased the performance, allowing us to double the processing speed compared to previously reported acoustic CD4 separation while still facilitating an efficient separation with fully preserved functional capacity of the sorted cells. Bead-mediated acoustophoresis can therefore be used to target and separate specific cell populations from a complex sample in a continuously perfused microfluidic device. Furthermore, the technology opens up the potential for simultaneous acoustic separation of several cell populations on a single device.
